# The Epidemiology and Mechanisms of Lifetime Cardiopulmonary Morbidities Associated With Pre-Pregnancy Obesity and Excessive Gestational Weight Gain

**DOI:** 10.3389/fcvm.2022.844905

**Published:** 2022-03-22

**Authors:** Shantanu Rastogi, Deepa Rastogi

**Affiliations:** ^1^Division of Neonatology, Children's National Hospital, George Washington University School of Medicine and Health Sciences, Washington, DC, United States; ^2^Division of Pulmonary and Sleep Medicine, Children's National Hospital, George Washington University School of Medicine and Health Sciences, Washington, DC, United States

**Keywords:** obesity, pregnancy, gestational weight gain (GWG), cardiac outcomes, pulmonary outcomes

## Abstract

Obesity has reached pandemic proportions in the last few decades. The global increase in obesity has contributed to an increase in the number of pregnant women with pre-pregnancy obesity or with excessive gestational weight gain. Obesity during pregnancy is associated with higher incidence of maternal co-morbidities such as gestational diabetes and hypertension. Both obesity during pregnancy and its associated complications are not only associated with immediate adverse outcomes for the mother and their newborns during the perinatal period but, more importantly, are linked with long-term morbidities in the offsprings. Neonates born to women with obesity are at higher risk for cardiac complications including cardiac malformations, and non-structural cardiac issues such as changes in the microvasculature, e.g., elevated systolic blood pressure, and overt systemic hypertension. Pulmonary diseases associated with maternal obesity include respiratory distress syndrome, asthma during childhood and adolescence, and adulthood diseases, such as chronic obstructive pulmonary disease. Sequelae of short-term complications compound long-term outcomes such as long-term obesity, hypertension later in life, and metabolic complications including insulin resistance and dyslipidemia. Multiple mechanisms have been proposed to explain these adverse outcomes and are related to the emerging knowledge of pathophysiology of obesity in adults. The best investigated ones include the role of obesity-mediated metabolic alterations and systemic inflammation. There is emerging evidence linking metabolic and immune derangements to altered biome, and alteration in epigenetics as one of the intermediary mechanisms underlying the adverse outcomes. These are initiated as part of fetal adaptation to obesity during pregnancy which are compounded by rapid weight gain during infancy and early childhood, a known complication of obesity during pregnancy. This newer evidence points toward the role of specific nutrients and changes in biome that may potentially modify the adverse outcomes observed in the offsprings of women with obesity.

## Introduction

In the last few decades, there has been a substantial increase in the incidence of obesity among the general population in developed countries. The >30% current prevalence of obesity is indicative of pandemic proportions (1). Similar trends are emerging from developing countries as well (2). The one subset of the population that has seen a disproportionate increase is women of child-bearing age among whom incidence of obesity is up to 50%, directly contributing to a higher proportion of pregnant women with obesity (3). Excessive gestational weight gain (GWG), which is strongly associated with pre-pregnancy obesity, further contributes to obesity during pregnancy.

Obesity during pregnancy is associated with immediate adverse perinatal outcomes in the mother and her newborns and long-term morbidities in the offsprings. Pre-pregnancy obesity and excessive GWG are also associated with a higher incidence of co-morbid conditions including gestational diabetes and hypertension, which independently contribute to perinatal as well as early-life complications among the neonates of obese women. Furthermore, along with higher incidence, more severe short-term and long-term morbidities among neonates compounds the risk of adverse long-term outcomes involving many organ systems, including the cardiovascular and pulmonary systems, which are affected as early as childhood and continue to be affected in young adulthood and later in life.

Multiple mechanisms have been proposed to explain the epidemiologic associations between obesity during pregnancy and adverse outcomes in the offsprings, several of which are related to the emerging knowledge of pathophysiology of obesity-related diseases in the adult population. These include changes in the endocrine-metabolic-immune pathways, associated with changes in the biome and with epigenetic alterations that directly influence RNA and protein expression. There is also evidence that these pathways continue to play a role during rapid weight gain in infancy in these offspring, which is an independent complication of obesity during pregnancy.

**Figure 1:**
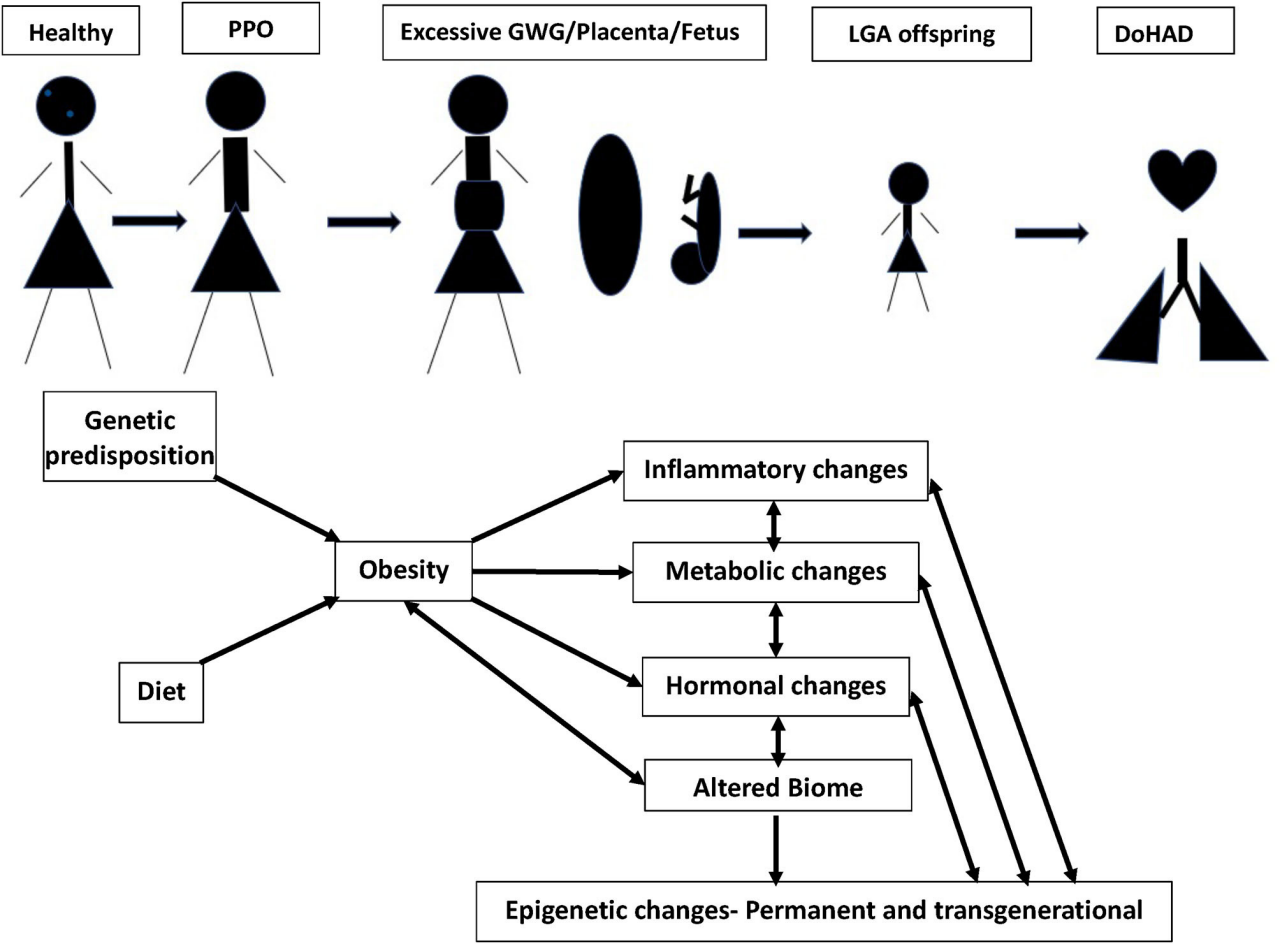
Continuum of adult obesity, PPO (pre-pregnancy obesity), with excessive GWG (gestational weight gain), causes changes in placenta and fetus, leading to birth of LGA (large of gestational age) offspring, potentially setting up for DoHAD (developmental origins of health and disease). The mechanisms by which PPO and GWG influence the placenta and developing fetus leading to an LGA neonate primed for cardiopulmonary disease development over the lifetime are summarized.

In this review, we discuss the associations of pre-pregnancy obesity and GWG with neonatal, infant, childhood, and adult outcomes, and the mechanisms underlying these associations (Figure 1). Although obesity affects many organs in both the women with obesity and their offspring, we focus this review on its cardiopulmonary effects.

## Cardiopulmonary Complications Among Offsprings of Women with Obesity During Pregnancy

### Pulmonary Complications

#### Newborns

Newborns of women with obesity during pregnancy present with complications as early as at the time of delivery, which include higher incidence of preterm delivery and pre-maturity-associated pulmonary complications (4). Some of the disease processes involving the offsprings' lungs are due to the direct effect of maternal physiology on lung development, and others are due to secondary complications such as prematurity, premature rupture of membrane, difficult birth process, fetal anomalies, and excessive or decreased fetal growth rates (5) which have independent effects on lung development and maturation.

Neonatal pulmonary diseases that are associated with obesity during pregnancy include the following:

1. Transient Tachypnea of Newborn (TTN): Higher incidence of TTN is observed among neonates born to women with obesity during pregnancy (6). TTN in neonates is related to a delay in maturation of endothelial sodium channel receptors which lead to decreased clearing of alveolar fluid. TTN may also be related to preterm delivery, maternal diabetes, or due to the changes in the steroid metabolism in the maternal-placental-fetal unit (5).
2. Respiratory distress Syndrome (RDS): There is higher incidence of RDS among the neonates born to women with obesity during pregnancy and is associated directly to higher incidence of preterm or indirectly to the obesity-related maternal co-morbidities (6, 7). Comorbidities such as gestational diabetes and pregnancy-associated hypertension play an additional role in delaying the maturity of the lungs, directly contributing to RDS, which is often observed in more mature neonates born late preterm and early term. One of the hypothesized mechanisms is the contribution of increased insulin production in the newborn to suppression of surfactant production. Further, changes in the glucocorticoid concentrations and higher occurrence of oxidative stress due to maternal obesity can worsen the presentation of the RDS in the newborns (8).
3. Chronic lung disease/Broncho-Pulmonary Dysplasia (BPD): Chronic lung disease of pre-maturity/BPD is a direct sequela of neonatal RDS. Occurrence of RDS, its management with mechanical ventilation, along with persistence of the inciting factors linked with maternal obesity such as systemic inflammation and oxidative stress directly contributes to the development of BPD (9).

#### Children/Adolescents

Obesity during pregnancy has also been associated with pulmonary morbidity during childhood as well as adolescence, including the following:

1. Asthma and hyperreactive airways: There is a linear relationship between childhood asthma and maternal body mass index (BMI) (10, 11). This observation is especially pronounced in non-atopic mothers, a detail that is of significance given the well-established association of atopy with childhood asthma. Longitudinal studies suggest that this relationship is usually established by 1 year of life and persists at least until 5 years, although the association at 5 years is not as strong as that observed during the first year of life (12). A recent meta-analysis demonstrated a longer impact of obesity during pregnancy, which was associated with a 31% increased risk of asthma or ever-wheezing in children aged 14 months until up to 16 years of age (13). Each 1-kg/m^2^ increase in maternal BMI led to a 3% increased risk of childhood asthma (5). Furthermore, excessive GWG was independently associated with a 16% higher risk of asthma or ever-wheezing, but not with current asthma or wheeze. In keeping with the association of obesity during pregnancy with non-atopic asthma in offspring, its association with other atopic disorders including allergic rhinitis, hay fever, atopic dermatitis, or inhalant and food allergen sensitization are inconsistent, suggesting that a mechanism different than atopy underlies the effect of maternal obesity on airway disease in their offspring. Future research is needed to elucidate these mechanisms.
2. Increased pulmonary infections. Maternal obesity is an independent risk factor for long-term infectious morbidity of the offspring (hazard ratio = 1.125) (14), with respiratory infections being the most common. In keeping with the effect of obesity on incident asthma, relative risk of wheeze, prolonged cough, and lower respiratory tract infection among offspring of women with obesity is also increased with relative risk (95% CI) of 1.09 (1.05–1.13), 1.09 (1.03–1.14), and 1.13 (1.07–1.20) for each 5 kg/m^2^ increase in maternal BMI (15).
3. Sleep disordered breathing: Although direct associations between obesity during pregnancy and sleep disordered breathing in children is not known, childhood obesity as a complication of obesity during pregnancy is significantly associated with sleep disordered breathing (16). Maternal obesity is associated with the large for gestational weight infants, who demonstrate rapid and sustained weight gain leading to an overweight /obese child among whom sleep disordered breathing is more common as compared to lean children (17).

#### Adults

While there are no direct studies on the pulmonary effects of obesity during pregnancy on adult offsprings, an association between obesity during pregnancy and COPD among their offspring in adulthood has been reported (18). It is ascribed to early environmental exposures, which lead to early development of chronic obstructive lung diseases through epigenetic influences such as altered DNA-methylation which in turn informed RNA expression patterns (18). In addition, obesity during pregnancy has been linked to changes in pulmonary function in adulthood, such as reduced FEV_1_, which may be a continuation/persistence of pathophysiological changes that started in early childhood into adulthood, due to asthma, or pulmonary infections, and possibly sleep disordered breathing associated with obesity during pregnancy. This speculation is supported by the independent effect of obesity during pregnancy on FEV_1_ in adult offspring, even after correcting for other factors, including adult weight. It is important to note that lower FEV_1_ was not linked to adult wheeze. This observation may be due to the known discrepancy between symptoms and pulmonary function, given that childhood wheeze is often in the presence of normal lung function (19), or it may be due to bias of the subjective nature of symptom report collection. One may also speculate that progressive but not acute deterioration of the lung function from infancy into adulthood may occur in absence of symptomatology supporting the hypothesis that processes that affect lung health at birth and early infancy persist and contribute to adverse effects on the lungs in adulthood (20). This concept forms the cornerstone of the developmental origins of health and disease (DoHAD) hypothesis. The hypothesis supports that changes that start early in life (21), due to early life adverse exposures including pre-pregnancy obesity, increased GWG, increased weight gain during infancy, and inadequate maternal diet during pregnancy and maladaptive development during fetal life and infancy (18), result in impaired lung growth with smaller airways and decreased lung volumes, leading to increased risk of bronchopulmonary dysplasia, asthma, or COPD, based on the age of the offspring at presentation of disease.

### Cardiovascular Complications

#### Newborns

Adverse cardiovascular (CV) effects have been observed in the neonates born to women with obesity during pregnancy, including higher systolic blood pressure, left ventricular mass, and aortic root diameter. In addition, children of women with obesity during pregnancy who often had rapid growth during infancy, have a 3-fold higher risk of an adverse cardiometabolic risk profile, which included high abdominal fat mass, high blood pressure, high insulin and triglycerides levels, and low HDL-cholesterol level at the age of 6 years (13).

Neonatal cardiovascular diseases that are associated with obesity during pregnancy include the following:

1. Structural cardiac effects: congenital heart disease (CHD**)** (22): Maternal obesity during pregnancy increases the offspring's risk to develop several different CHDs, including transposition of great arteries, aortic arch defects, single-ventricle heart, atrial septal defect, patent ductus arteriosus, and pulmonary valve defects, in a dose dependent manner (23). Obesity alters endothelial cell function, causing chronic cell activation, as well as upregulation of pro-inflammatory immune responses (24–27). Endothelial cell dysfunction in mice during embryogenesis has been shown to cause CHD (24, 28, 29). While physiologic levels of insulin and adiponectin activate endothelial nitric oxide synthase (30, 31), that increases nitric oxide (NO), a regulator of vascular tone, insulin resistance and reduced adiponectin in the context of obesity diminish endothelial cell production of NO. This reduction in NO and maternal obesity associated abnormalities in placental vascular supply, alter fetal vascular circulation, directly impacting embryogenesis (32). Effects of endothelial cell dysfunction may persist after birth, as observed among the offspring of non-human primates exposed to a high-fat diet during pregnancy (33). Other mechanisms proposed to contribute to the development of CHD are changes in left-right patterning (34), increased apoptosis resulting from oxidative stress (35, 36), and alterations in cell formation, and cell migration (36). Difficulty in performing antenatal echocardiogram due to maternal obesity is another contributory factor leading to missed antenatal and higher postnatal diagnosis of CHD (37–39).
2. Non-structural cardiac effects: Neonates of women with obesity during pregnancy have been observed to have higher systolic blood pressure (40). Both pre-pregnancy obesity and excessive GWG directly contribute to higher fasting glucose levels and negatively correlate with HDL-cholesterol levels and glucose-to-insulin ratio of neonates, regardless of neonatal adiposity (41). Given the limited reporting of these effects, the differential effect of pre-pregnancy obesity and that of GWG is also not well-understood. Other non-structural cardiac effects have been observed among children but not among neonates of women with obesity during pregnancy.

#### Children/Adolescents

CV risk in offspring: Cardiovascular effects of maternal obesity in pregnancy among offsprings in their childhood/adolescence is best described in the context of persistent obesity in childhood, which increases by 3% for every 1 kg of maternal GWG (42). Both pre-pregnancy obesity and GWG have been associated with higher systolic and diastolic blood pressure in early and late childhood, and altered cardiometabolic profile, including insulin resistance and low HDL cholesterol levels (43). Further, both early onset and excessive GWG are directly linked to higher risk of an adverse cardiometabolic profile in childhood (44–46).

Other changes observed in the offsprings of women with obesity have been related to the alterations of microvasculature, which regulates blood flow to the capillary bed and thereby regulates nutrition and growth to the organs. The microvasculature in neonates born to mothers with obesity have fewer number of vessels that have a more simple branching pattern with decreased ability to vasodilate at times of increased demand for blood supply to the tissue (47). Inability to vasodilate to meet the demands of the organ are related to the lack of production of NO, prostaglandins and endothelium derived hyperpolarization factors which are effected by inflammation, oxidative stress (48) and changes in the gene function (49). These changes have been demonstrated in murine and non-human primates, and more recently these were demonstrated as an abnormality in the retinal microvasculature of children born to women with obesity (50).

#### Adults

Higher pre-pregnancy obesity and higher GWG have been independently associated with a higher percentage of body fat in the offspring at the age of 30 years (51), a finding that has been validated in subsequent studies (52, 53). In a long-term prospective longitudinal study, the authors demonstrated that pre-pregnancy obesity was associated with higher BMI in offspring at all ages, from childhood until adulthood, suggesting that obesity during pregnancy is a persistent risk factor for lifelong obesity (54). GWG is also independently associated with higher systolic blood pressure in the offspring at 21 years, with 0.1 kg/week increase of GWG in the mother associated with 0.2 mmHg higher systolic blood pressure (55). Animal studies have facilitated an understanding of the mechanisms by which obesity during pregnancy increases cardiometabolic complications in the offspring. Litter born to rats fed an obesogenic diet during pregnancy had higher weight and BP (34), due to increased sympathetic drive during early development, because of altered leptin signaling. Oxidative stress, and proinflammatory cytokines, modulated by the high fat diet also play a role in alteration of vascular tone. Further, the renin-angiotensin system is also altered leading to augmented effects of angiotensin II and TNF-α in the brain, which in turn influence angiotensin II-elicited hypertensive response in adult offspring (56).

## Pathogenesis of Cardiopulmonary Effects in the Offspring of Women with Obesity During Pregnancy

Having summarized the cardiopulmonary complications in offspring of women with obesity during pregnancy, we discuss the mechanisms that underlie these associations under the following four subheadings, (a) role of immune and metabolic effects, (b) role of oxidative stress, (c) role of microbiome, and (d) role of epigenetics. Although they have been discussed individually, we would like to emphasize that these different mechanisms are intertwined and have strong interactions between them (Figure 1).

### Immune and Metabolic Effects

#### Immune Effects

**Figure 2:**
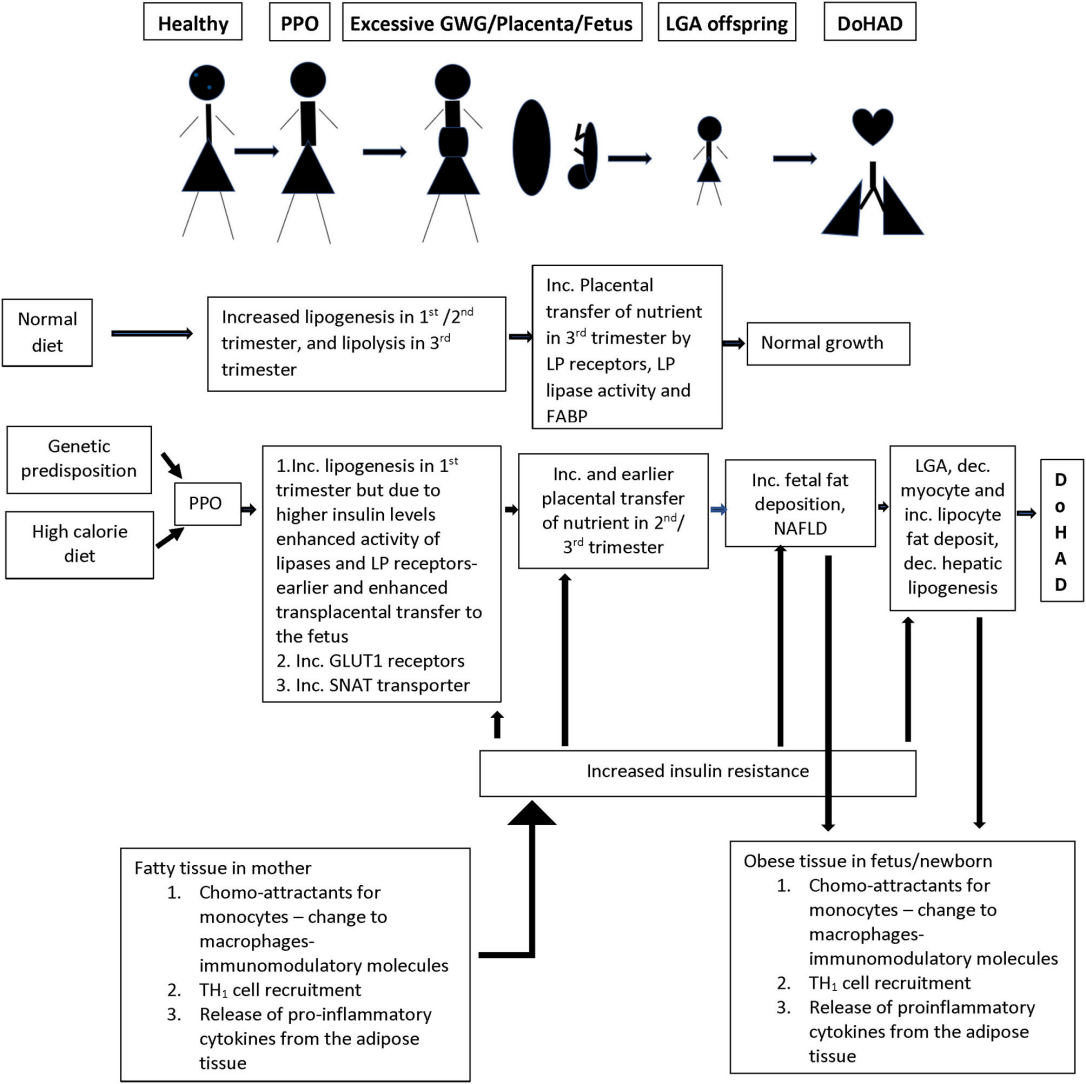
Summary of the changes in the lipid/energy metabolism in a normal pregnancy as compared to nutrient/inflammation related changes in women who develop PPO (pre-pregnancy obesity) with or without GWG (gestational weight gain). There is a complex interaction between inflammation in adipose tissue and nutrient metabolism in pregnant women, and the transfer of nutrients through the placenta to the developing fetus, which is associated with increased growth and birth of a LGA (large for gestational age) infant who persists with similar nutrient/ inflammatory changes as in the mother creating an environment for the development of short- and long-term changes in the cardiorespiratory system and associated disease. LP, lipoprotein; FABP, fatty acid binding protein; GLUT1, glucose transporter 1; SNAT, system A amino acid transporter.

There is increased TNFα secretion in adipose tissue from women who develop obesity during pregnancy with or without co-morbidities such as diabetes mellitus/ gestational diabetes mellitus (57) (Figure 2). Adipose tissue macrophages play a sentinel role in adipose tissue expression of genes that encode inflammatory proteins, including interleukin −6 (IL-6), monocyte chemotactic protein −1 (MCP-1), inducible nitric oxide synthase (iNOS), and matrix metalloproteinases (MMPs) the concentrations of which correlate with maternal adiposity. These macrophages are recruited from the bone marrow to adipose tissue in response to MCP-1 or chemokine ligand −2 (CCL-2) expression by adipocytes, which attract circulating monocytes with chemokine receptor-2 (CCR2) on their cell surface (58). These circulating monocytes adhere to the activated endothelium using integrins, Intercellular adhesion molecule 1 (ICAM1) and vascular cell adhesion molecule 1 (VCAM1), which are also increased in obese adipose tissue (59). After recruitment to the adipose tissue, monocytes differentiate into macrophages in response to macrophage colony stimulating factor (M-CSF), and once activated as M1 macrophages, produce immune modulatory molecules such as TNFα, IL-6, and those detailed above. These pro-inflammatory cytokines and chemokines activate intracellular signaling via transcription factors, including nuclear factor kappa-light-chain-enhancer of activated B cell (NF-κB), by inhibiting its inhibitory regulators (IκB1 and IκB2), setting up a positive feedback loop with increased release of TNFα and MCP-1 (60). Activation of serine/threonine kinases [c-Jun N terminal kinase (JNK)1, JNK2, and JNK3], that are upstream of NF-κB, additionally enhance inflammation (61). In addition to these proinflammatory proteins, adipose tissue also releases adipokines, including leptin and adiponectin. The inflammatory gene expression by adipose tissue macrophages is dynamic and modulated by changes in body weight (62). These cellular processes are also directly related to alterations in carbohydrate and lipid profiles associated with insulin resistance in women with obesity, which then sets up a positive feedback cycle between metabolic derangements and adipose tissue inflammation (59) (Figure 2). Epigenetic changes in the FTO gene, the gene most consistently associated with obesity, have been reported to play a role in the relationship between metabolic derangements and adipose tissue inflammation (63).

#### Metabolic Effects

##### Fat Metabolism

Fat metabolism plays a key role in the links between metabolic changes and adipose tissue immune responses during pregnancy. During normal pregnancy, there is a shift from lipogenesis to lipolysis between second and third trimesters with increase in adipose tissue low density lipoprotein (LDL) and triglyceride levels due to increased lipase activity associated with increase in insulin sensitivity that occurs in the first two trimesters. During the third trimester, there is increasing insulin resistance with enhanced lipolytic activity leading to increased free fatty acid (FFA), glycerol, and VLDL levels which are transported to the growing fetus (64) (Figure 2). In pregnancy complicated with obesity, the inflamed state of the adipose tissue directly feeds into earlier onset and higher levels, of insulin resistance, leading to earlier switch from lipogenesis to lipolysis during pregnancy. This earlier switch directly contributes to longer exposure of the fetus to higher levels of free fatty acids (FFA) and carbohydrate metabolites (65) (Figure 2).

The placenta plays an active role in transfer of maternal lipoproteins, especially essential fatty acids and long chain fatty acids, by utilizing receptors for lipoproteins, lipase activity, and fatty acid binding protein, and preventing passive transfer (66). Similarly, although cholesterol can be produced in the fetus, there is an efficient transport mechanism in the placenta transferring maternal cholesterol to the fetus. These placental transport processes are increased in the setting of higher circulating insulin levels in pregnancy complicated with obesity (65) related to increase in placental lipoprotein receptors which increase uptake of maternal VLDL and dietary chylomicrons, that are hydrolyzed by placental lipoprotein lipase and transported to the fetus (Figure 2). The changes in placental uptake and metabolism of lipids are associated with upregulation of nutrient sensing pathways such as mammalian target of Rapamycin (mTOR) and peroxisome proliferating activator receptors (PPAR) pathways driven by increased insulin in maternal circulation (65, 66) and are known to have gender-related differences on nutrient transport (66). Placental transport is further upregulated when there is coexisting maternal diabetes. Increased placental triglycerides contribute to a placental proinflammatory state with increased TNFα and interleukins (26). This pro-inflammatory state of the placenta is directly transferred to the growing fetus, particularly in the liver, which serves as a gateway for nutrients and chemicals in the fetus. There is increase in liver X receptors, in intrahepatic lipogenesis leading to increased lipid content in the liver, and development of non-alcoholic fatty liver disease (NAFLD) (67) which is further enhanced by excessive transplacental transport of the lipids (Figure 2). These complications are worsened by decrease in fatty acid oxidation.

In addition, there is increased FFA in the muscle of the fetus of women with obesity during pregnancy, with decreased myocytes and increased lipocytes (68). Higher muscle FFA concentrations increase serine phosphorylation of the insulin receptor, preventing insulin action, leading to worsening insulin resistance, and decreased glucose uptake by the muscles, with decreased hepatic output, further aggravating the cycle of lipolysis, and its associated inflammation and oxidative stress (69).

These immunometabolism profiles of fetuses of women with obesity during pregnancy have been directly linked to neonatal inflammation. Elevated IL-1β, IL-8, and MCP-1 in neonatal lungs have been associated with increased lung compliance and thereby better lung function due to its effect on accelerated surfactant production (70). However, inflammation also leads to disrupted alveolar septation and capillary development and disordered a-smooth muscle actin (myofibroblast marker) and elastin deposition in alveolar septa which contributes to development of BPD (70, 71). These diverse effects of inflammation are directly related to the fetal developmental stage, having a greater effect when they occur early as compared to later in pregnancy. The changes due to fetal inflammation in the context of maternal obesity have been reported to be similar to those seen in infants on mechanical ventilation for a period longer than 72 h (72).

##### Glucose Metabolism

In normal pregnancy, there is net glucose transfer from the mother to the fetus across the placenta *via* a family of facilitated glucose transporters (GLUTs), that increase in density in the first half of the pregnancy. These receptors play differing roles in glucose transport with GLUT1 receptors being mainly responsible for the transport across the basal membrane in the placenta (65). About 75% of the glucose passes unchanged and the rest is either utilized or changed to glycogen or lipids. This lipogenesis is directly altered by changes in maternal glucose metabolism (73), due to changes in insulin levels, as detailed above (Figure 2). In the context of obesity during pregnancy, there is upregulation of GLUT1 expression. Since TNFα is associated with decreased glucose uptake and lipogenesis in the mother, higher TNFα in mother and GLUT1 receptors on the placenta directly increase the proportion of glucose transported across the placenta. However, the extent of glucose transfer in the pregnant women with diabetes is not simply explained by the increased glucose in their blood (74, 75), suggesting that there are yet undefined mechanisms that contribute to alteration in glucose transfer from mother to fetus in the context of obesity during pregnancy.

##### Amino Acid Metabolism

In normal pregnancy, amino acid transport is tightly controlled and is energy dependent against the concentration gradient (76). Many transport systems have been identified, and each amino acid may use one or multiple transporters and one pathway could be used for transport of multiple amino acids. In obese pregnant women, there is upregulation of the System A Amino acid transporter (SNAT), that increases neutral amino acid transport, which is associated with excessive fetal growth (77). SNAT levels correlate with maternal levels of IL-6 and TNFα, and their expression may involve insulin, mTOR, and Signal transducer and activator of transcription 3 (STAT3) pathways. The amino acids transported by this pathway are found to correlate more strongly with birth weight than maternal glucose levels (65), suggesting that their contribution of neonatal weight is independent of glucose metabolism.

### Oxidative Stress

Maternal obesity is also linked to increased oxidative stress, which may be due to mitochondrial processing of the high blood lipid content, leading to excessive generation of oxygen free radicals (78). In addition to circulating lipid content, fatty acid oxidation in the mitochondria in the muscles releases reactive oxygen species and increases oxidative stress. Mitochondrial injury due to maternal obesity and its effect on compromised metabolism has been reported even prior to conception with presence of oxidative stress in the ova (79).

The placenta produces pro- and anti- oxidants to keep lipid peroxidation under control. When exposed to excessive circulating levels of lipids, as seen in obesity during pregnancy, the placenta, which is rich in mitochondria, highly vascularized, and exposed to high maternal oxygen partial pressure, further contributes to increased superoxide production. Nitric oxide (NO) is also locally produced by the placenta from L-arginine, by NO synthase activity (80, 81). The tightly controlled production of these two free radicals, superoxide, and nitrosyl ion, by antioxidant defense system during normal pregnancies, goes awry in obesity during pregnancy. Obese pregnant women have high levels of oxidative stress, evident by high levels of malondialdehyde and carbonyl proteins, end products of oxidation, despite elevated levels of antioxidants including reduced glutathione and antioxidant enzymes, which together suggest that production of pro-oxidants far exceeds that of antioxidants (79).

Increased oxidative stress in offsprings of women with obesity during pregnancy correlates with degree of maternal obesity. There is increase in malondialdehyde, superoxide anion, and nitric oxide levels in newborns of mothers with obesity compared to those born to normal-weight mothers (82). In keeping with the maternal changes, there is a parallel increase in antioxidant enzymes, catalase, and superoxide dismutase, in the fetus as well, suggesting that over expression of fetal antioxidant activities is an adaptive response to counter the effect of increased oxidative stress (79, 82). However, there is little correlation between neonatal and maternal oxidative stress measures (82) supporting the protective modulation by the placental unit, and the likelihood that the newborn is mounting its own oxidative stress responses. Oxidative stress and its associated tissue damage have been related to adult-onset CV diseases among offspring of women that were obese during pregnancy, by potentially influencing endothelial changes, directly or *via* epigenetic effects by alteration of DNA (83). While these are the best understood mechanisms by which obesity during pregnancy contributes to disease among the offspring, further research is needed to elucidate the dynamic nature of the associations in the offsprings' lifetime.

### Biome

There is limited human data on the association of maternal biome with outcomes in the offspring, and most of the existent knowledge is from murine models. The importance of the gut biome has stemmed from its role in production of essential nutrients, prevention of colonization with pathogenic bacteria, and modulation of host metabolism by energy extraction (84), immunity (85), and lipid metabolism (86). Excessive weight gain in mice is associated with changes in proportion of Firmicutes to Bacteroidetes which are affected by differences in fat and carbohydrate dietary intake (87, 88) as well as by genetics (89), serving as a classic example of gene by environment interaction. Similar changes have been recently described in the women with obesity before the pregnancy and those who had excessive gestational weight gain where the bacterial colonization was different (90) and with lower diversity (91) than that in the lean women. These changes in bacterial proportions in turn influences the ratio of energy harvesting. Certain diets, referred to as “epigenetic diets,” contain methyl donors (e.g., folate and vitamin B12), green tea polyphenols, soybean isoflavone, and broccoli sprouts sulforaphane (92), and are metabolized differently by the gut microbiota. For example, Bifidobacterium strains can facilitate production of bioactive metabolites such as folate, butyrate, biotin, acetate, and acetyl-CoA in the human intestine, (93) which directly play a role in regulation of epigenetic processes, given the effect of methyl donors on DNA methylation and of butyrate, a histone deacetylase (HDAC), that can influence histone modification processes. Butyrate production is known to influence metabolism by epigenetically regulating obesity-related gene expressions such as PPARγ and IFNγ (92). As supporting evidence of overlapping mechanisms between obesity, gut biome, and epigenetic regulation, probiotic supplementation during pregnancy in a pilot human study was associated with alteration of DNA methylation of promoters of genes associated with obesity and weight gain both in mothers and their children (94) (Figure 1).

The fact that the placenta and intrauterine environment is not sterile and a microbiome exists in the placenta, amniotic fluid, umbilical cord blood, and meconium in healthy gestation, is additional evidence for the role of biome, and supports the “*in-utero* colonization” hypothesis (95). There is also evidence that mothers transmit microbiota to their offsprings in the *in-utero* environment prior to birth (95, 96). The extent to which this early exposure to a pathological biome influences cardiopulmonary outcomes in the offspring is not known. There is controversy about the transference of microbial species from the mother to the fetus and their role in nutrient metabolism. For instance, metabolites of isoflavones transfer from mother to fetus through the placenta, are further metabolized and excreted through microbiota on the fetal side of the placenta, and are released in the amniotic fluid (97). Thus, it is possible that the offspring can be colonized by microbial species from the mother *in-utero* to help them efficiently metabolize nutrients that benefit their future health. However, the transmission route, the mechanism of metabolism in the fetus, and the effects of fetal exposure to bioactive diets, needs further investigation.

Maternal diets can also influence gut microbiota composition in the offspring through maternal gut microbiota transfer. These changes have been reported to persist until adulthood and exert long-term effects on metabolic health and disease development (92). The gut microbe-mediated epigenetic changes in the host are proposed to be due to specific changes in patterns of epigenetic modifications, highlighting the complexity of interactions between the microbiome, metabolome, and epigenome (98). In addition to intrauterine period, the postnatal stage during lactation is also important for establishment of gut microbiota composition and can be influenced by several factors such as contact with the mother, maternal diets, and breastfeeding/formula (99). In non-human primates, maternal high fat diet was associated with reduced abundance of protobacteria, e.g., Campylobacter species in the offspring gut compared to the control diet group, even though the primates were weaned from high fat diet and switched to control diet in few weeks; this diet change related effect on the biome alteration persisted for 6 months (100). This study provides early evidence that maternal diet can shape the commensal microbiome profiles for a long duration or even permanently in the offspring's gut. We speculate that original seeding of the microbiome from maternal transmission may play profound roles in influencing development of adult biome and diseases in later life. Cross-fostering studies in mice born to the normal diet-fed mother but breastfed by a high fat diet-fed mother have supported this speculation highlighting the importance of maternal diet during both pregnancy and lactation to offspring health (101).

In addition to maternal diet, the method of delivery contributes to early microbiome establishment. Babies delivered by cesarean section (C-section) acquire a microbiota that differs from that of vaginally delivered infants (102). C-section delivery is associated with delayed acquisition of beneficial bacterial colonization such as Bifidobacterium in the newborn guts (103), which may directly contribute to increased risk of obesity in later life. Others have shown differing bacterial colonization in newborns born to women with obesity or increased gestational weight gain, that do not correlate with the maternal gut flora, but are different from normal flora and have less diversity compared to newborns of women with healthy weight (91). This may be due to differing reasons, such as the need for C-section, use of antibiotics, and absence of breast feeding, and the authors have suggested that furthering these studies beyond taxonomic classification to those based on metagenomic sequencing will better elucidate their functions (90). Although these studies have begun to highlight the importance of maternal biome in immediate and long-term health effects in the offspring, there is much research that needs to be done to facilitate a complete understanding of the links between maternal diet, lipid, glucose, and AA metabolism, fetal and newborn biome, and childhood and adult-onset diseases.

### Epigenetic Changes

Epigenetic changes including DNA methylation, histone modification, and micro-RNA expression, associated with metabolic and inflammatory markers, can persist for multiple generations and influence health and disease. Such changes have been observed in the placenta and the newborns of the obese women (104). DNA methylation is the best studied of these mechanisms and DNA methylation of gene promoters has consistently been associated with gene silencing. The association of diet with methyl group donors is particularly critical in an adverse nutritional environment such as obesity during pregnancy (105); some of these details have been discussed under the metabolic and immune and biome sections. Generalized intrauterine malnutrition has been linked with genome-wide changes (106). Histone modification is the other epigenetic mechanism that has been associated with short term/flexible changes in the gene expression. For instance, acetylation of the lysine residues on the tails of the histone correlates with active chromatin and gene transcription. Histone acetylation due to maternal high fat diet has been associated with sustained inflammatory response in the vascular smooth muscle cells (106). Maternal high fat diet in primates can modulate fetal type III histone deacetylase, sirtuin1, which links to regulation of the fetal epigenome and metabolome (107). In rodents, maternal high fat diet was associated with decreased adiponectin and increased leptin gene expression partially due to an increased enrichment of the transcriptional activator in the promoter of the leptin gene (108). MicroRNAs have also been linked to temporal control of gene expression during normal development and have been implicated in chorioamnionitis and maternal infection (109).

Furthermore, metabolic biomarkers have been linked to changes in epigenetic marks in the placenta of women with obesity. Up to 67% of differentially methylated genes in the placenta were explained by high maternal TG levels and 9% due to high maternal glucose levels. These changes in DNA methylation may have varied effects on the placenta, including increased inflammation, associated with increased gene expression of leptin and TNFα due to the association between metabolic and immune responses.

Differential gene DNA methylation is particularly important in the fetus because of its role in cell differentiation and organogenesis. Therefore, changes in DNA methylation due to changes in metabolism, biome, or related to inflammation, can alter development and maturation of any organ system. Alterations in programming prior to the final differentiation of cells could directly present as different cellular phenotypes from a single genotype, depending on when the change occurred (110). If the change persists until the final maturity of the call, phenotypical change can be permanent. While some of these changes have no adaptive advantage, others allow the fetus to respond and adopt to immediate environment for self-preservation, and others produce predictive adaptation which have no obvious immediate value but occur in expectation of altered future environment, the latter being maladaptive and associated with adult onset disease (106). One such example of epigenetic change is suppression by poly unsaturated fatty acid of the leptin gene among the newborns of women with obesity which decreases the normal function of leptin, that increases satiety. Lack of leptin is then associated with increased intake, proinflammatory activity, and obesity in the offspring (111). Another epigenetic effect of high free fatty acid in the offspring of women with obesity is the silencing of homeobox gene Pdx-1 which increased islet cell apoptosis and worsens the failing hyperplastic islet cells in newborn observed in women with obesity, setting the stage for early pancreatic exhaustion and type 2 diabetes in the offspring of women that were obese during pregnancy (68). Based on Extremely Low Gestational Age Newborn Study (ELGAN study), inflammation and abnormal placental transport are two mechanisms of epigenetic programming associated with maternal obesity that increase the risk for poor outcomes (112). As summarized for the biome, the literature on the role of epigenetics in adverse outcomes in offspring of women with obesity during pregnancy is sparse; therefore, there is a need for more research in this field.

### Focused Discussion of Difference Between Gestational Weight Gain and Pre-Pregnancy Obesity

Given the varied effects of obesity on metabolism, which in turn influences immune, biome and epigenetic responses, we would like to highlight the importance of the timing of weight gain during pregnancy and distinguish between pre-pregnancy obesity and excess GWG with regards to outcomes in the offspring.

While both pre-pregnancy obesity and GWG have a linear relationship with poor perinatal outcomes and newborns' birth weight, the effect is more pronounced among women with pre-pregnancy obesity as compared to those with excessive GWG. Pre-pregnancy obesity explains up to 24% of complications in the newborns. Large for gestational age (LGA) complication, to which GWG contributes up to 32%, is the only complication where GWG contributes to a higher extent than pre-pregnancy obesity. While the correlation of GWG with LGA status is stronger among nulliparous women (*r* = 0.26) than among multiparous women (*r* = 0.16), this association is lost among women with pre-pregnancy obesity (113). Conversely, although both pre-pregnancy obesity and excess GWG contribute to adverse outcomes in offspring, excessive GWG among women with pre-pregnancy obesity further worsen the outcomes (114). However, this correlation becomes weaker when the woman is already >135% of ideal weight for height before conception, suggesting a dynamic association between pre-pregnancy obesity and excess GWG.

The effect of pre-pregnancy obesity on the fetus's body fat content is probably related to alteration of placental functions at conception, especially related to early exposure to high levels of maternal cytokines and altered lipid gene functions, associated with altered lipid metabolism, and increased transport, during the latter part of pregnancy. This observation is further supported by the fact that weight reduction among women with pre-pregnancy obesity before conception was associated with less decrease in insulin sensitivity during pregnancy and with lower newborn body fat content as compared to women with pre-pregnancy obesity who lost weight during pregnancy (115). These temporal associations highlight the importance of the dynamic effect of hormonal- metabolic milieu changes on fetoplacental growth, particularly since obesity at onset of pregnancy is associated with increased transport of calories across the placenta for the entire duration of pregnancy (116). In keeping with the importance of the timing of weight gain/loss in association with pregnancy, the timing of excessive GWG relative to the trimesters is also critical as excessive GWG in early pregnancy is more strongly associated to the increasing BW and obesity in childhood (13).

Weight distribution is also associated with altered metabolic profiles. Non-obese women accumulate fat in the lower body during pregnancy while women with obesity accumulate fat in the upper body (117). Upper-body obesity is associated with reduced uptake and storage of fatty acids, along with increases in lipolysis (118). In contrast, lower-body fat accumulation is associated with more-favorable lipid and carbohydrate metabolic regulation (119) and an overall lower-risk metabolic profile. Fat distribution also influences co-morbid conditions. Diabetes, one of the complications of differential fat distribution, is common in mothers with obesity, and is known to induce pro-inflammatory genes in the placenta (116). Thus, body fat distribution additionally contributes to the negative effects of adverse metabolic changes in obese individuals, which directly contribute to adverse fetal outcomes.

It is important to highlight that the size and fat content in neonates is determined by maternal rather than paternal anthropometry, which has minimal effect (120). Fat distribution is also different in offspring of women with pre-pregnancy obesity as compared to mothers who were lean before pregnancy. Newborns of women with pre-pregnancy obesity have central distribution of subcutaneous fat, as compared to newborns of women lean before pregnancy, where fat accrual is more peripheral (biceps and triceps area). This has direct implications on insulin sensitivity since central fat deposits are consistently associated with increased insulin resistance and associated with more inflammation as compared to deposits in peripheral muscles (113). These differences also influence long term effects, where the relationship is more complex, and have a J-shaped association. The chances of overweight/obese status is increased among those born SGA as well as those born LGA (121). LGA is also more predictive of metabolic syndrome at 11 years with hazard ratio of 2.6. However, the association of pre-pregnancy obesity and excess GWG is less clear on the development of adult obesity, which is also influenced independently by early-life weight gain (122). These findings highlight the complex and dynamic nature of weight change among women of childbearing age and their influence on early and late health outcomes in their offspring.

The importance of pre-pregnancy weight and weight gain during pregnancy highlights the need for management approaches for weight gain during pregnancy. In addition to early recognition of the problem and planning for pre-pregnancy weight reduction, it is pertinent that recommendations of Institute of Medicine regarding weight gain criteria for the obese mothers and post neonatal period follow up of their newborns for long term outcomes (123) be followed closely.

## Conclusions and Areas of Future Research

Although current evidence suggests that maternal obesity during pregnancy adversely affect common childhood health outcomes, there are several areas in need of future research. Despite extensive adjustment for potential confounding factors in most studies and the use of more sophisticated study designs in observational studies, the causality of the observed associations remains unclear. Future studies need to move beyond childhood adiposity as an outcome of interest and focus on overall outcomes such as cardio-metabolic, respiratory, and cognitive outcomes. Long-term follow-up of participants in trials focused on reducing maternal weight throughout pregnancy will also provide much needed insight into the causality.

Future progress is based on better understanding of the mechanisms, identifying areas of intervention, early recognition of the long-term adverse outcomes in the offsprings, and interventions to change or prevent the course for these poor outcomes. Although animal studies and few human studies have suggested several pathways that may be involved in the observed associations, these pathways need detailed investigation especially in human studies. Future studies are also needed regarding detailed assessments of the maternal exposures of interest for the duration of pregnancy, such as repeated measurements of maternal weight, body composition, and metabolic status. Similar details are also needed for outcomes in offsprings, with inclusion of more detailed objective and repeated measures of organ system structure and function during the life course in addition to growth and adiposity parameters. Since epigenetic mechanisms are one of the major underlying mechanisms of interest, repeated offspring blood samples throughout the life course are needed to assess the influence of the specific maternal exposures on the offsprings' epigenetic adaptations. These studies together will identify modifiable aspects of pre-pregnancy obesity and excessive GWG for prevention of adverse health outcomes in the offspring. Improved understanding of the mechanisms underlying complications of obesity during pregnancy in conjunction with execution of IOM guidelines for weight management during pregnancy will have a life-long impact on future generations.

## Author Contributions

SR and DR contributed to the concept and development of this manuscript. All authors contributed to the article and approved the submitted version.

## Funding

DR was funded by NIH grant HL141849.

## Conflict of Interest

The authors declare that the research was conducted in the absence of any commercial or financial relationships that could be construed as a potential conflict of interest.

## Publisher's Note

All claims expressed in this article are solely those of the authors and do not necessarily represent those of their affiliated organizations, or those of the publisher, the editors and the reviewers. Any product that may be evaluated in this article, or claim that may be made by its manufacturer, is not guaranteed or endorsed by the publisher.
